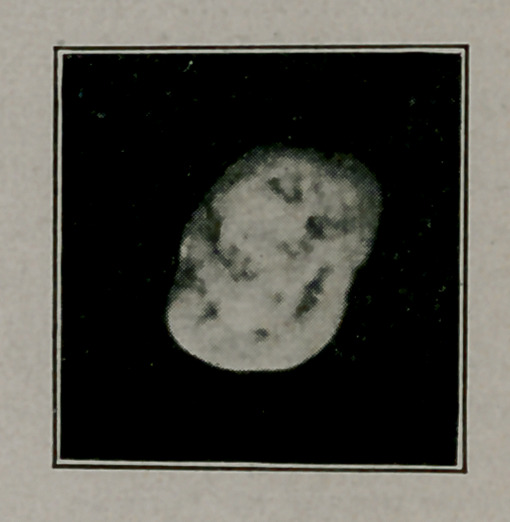# Foreign Bodies of the Trachea, Bronchi and Oesophagus

**Published:** 1915-12

**Authors:** 


**Foreign Bodies of the Trachea, Bronchi and Oesophagus.** Robert L. Moorehead, Brooklyn, L. I. Med. Jour., Oct. Space does not allow the details of diagnosis and methods of extraction. The pictures speak for themselves.

## Figures and Tables

**Figure f1:**
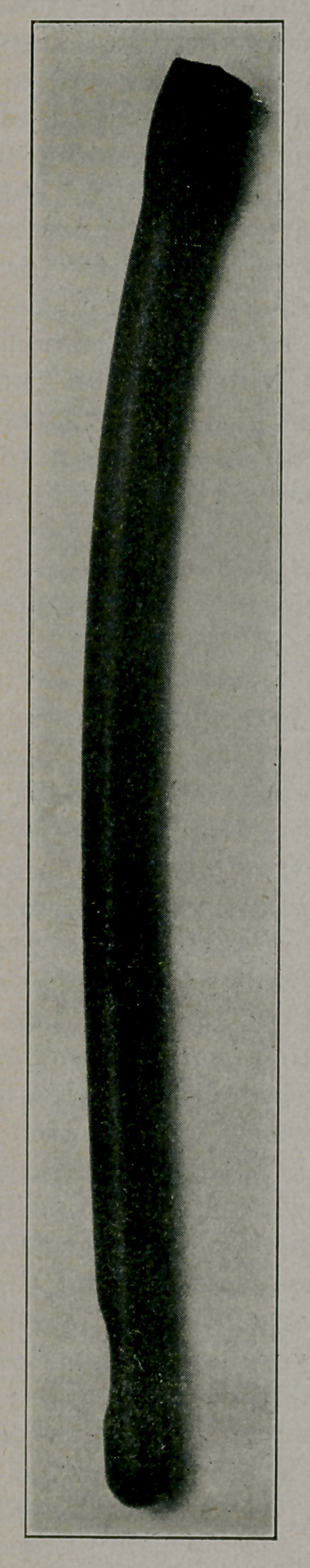


**Figure f2:**
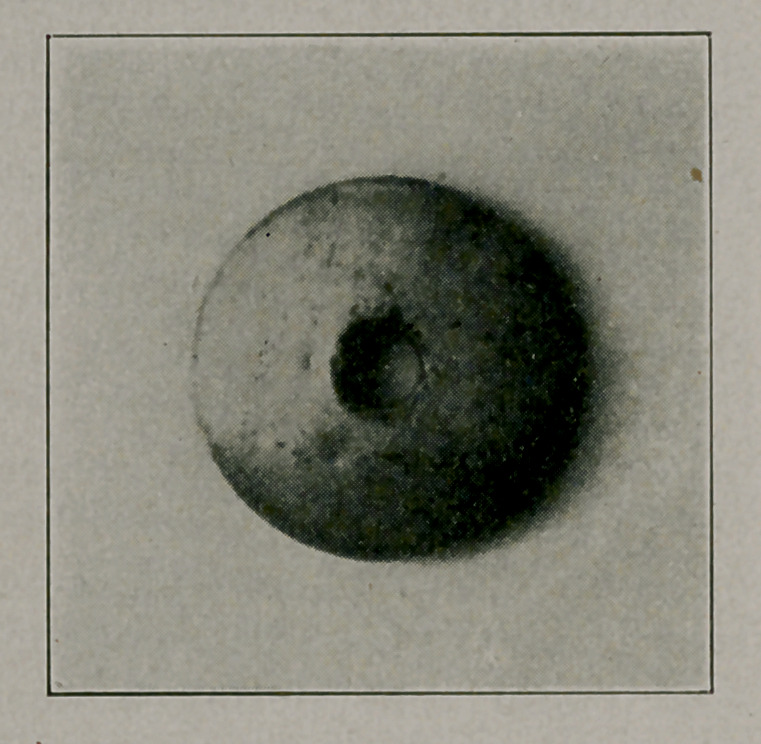


**Figure f3:**
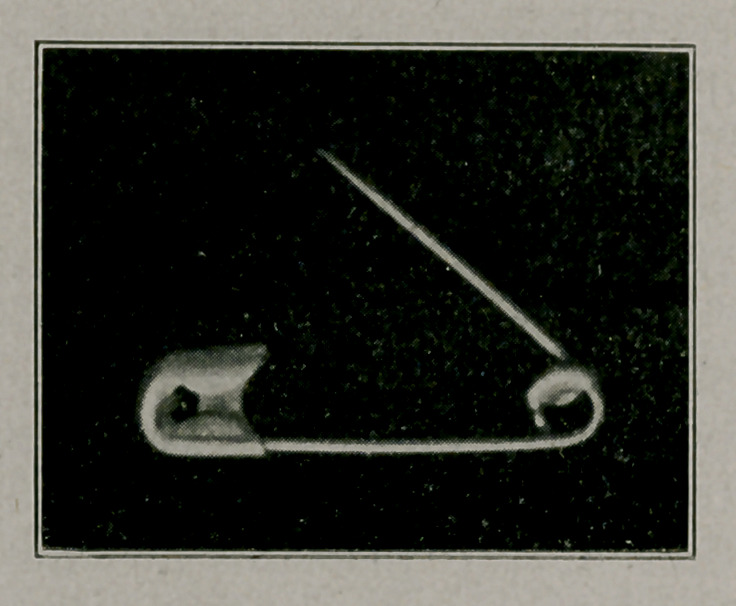


**Figure f4:**
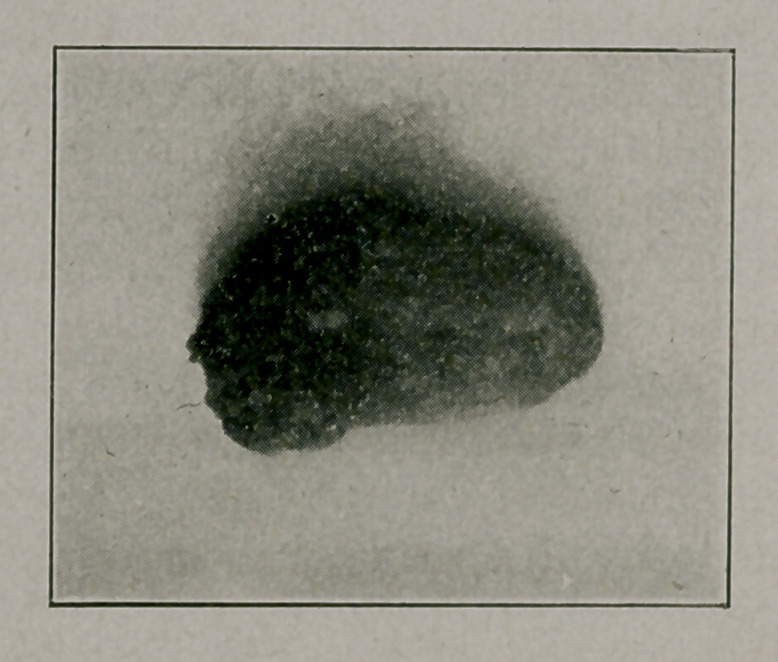


**Figure f5:**
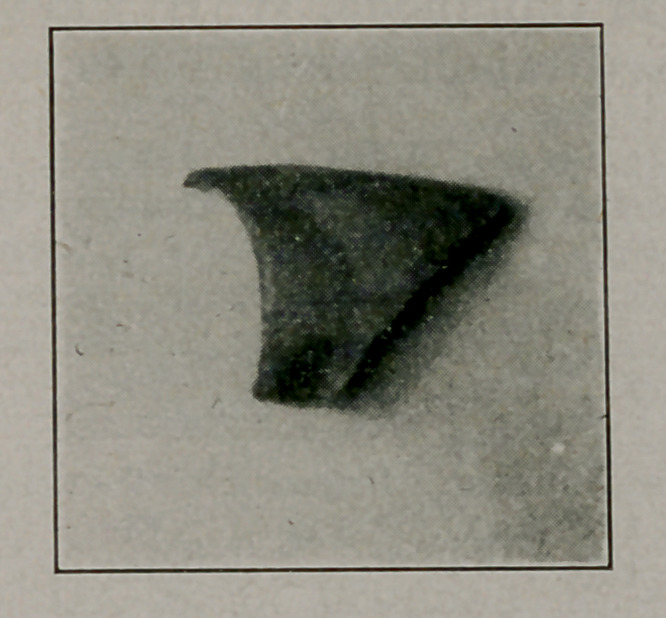


**Figure f6:**
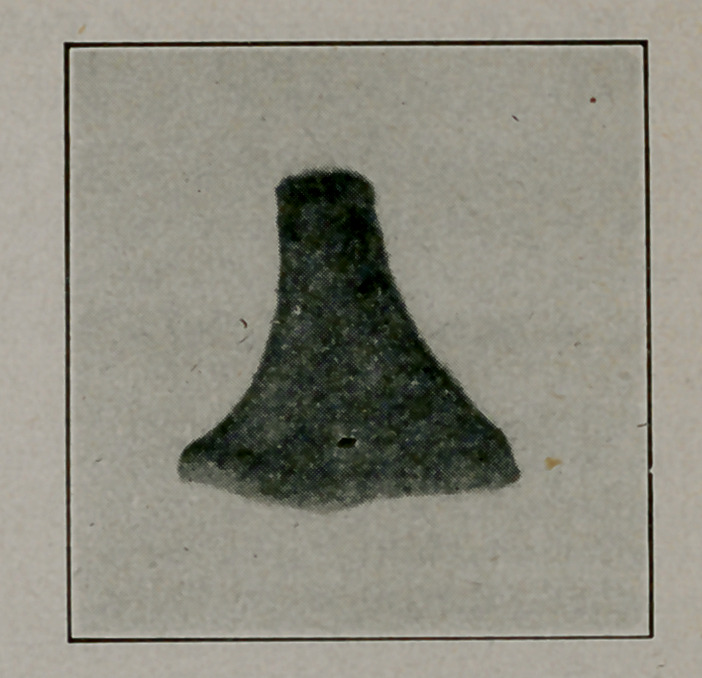


**Figure f7:**
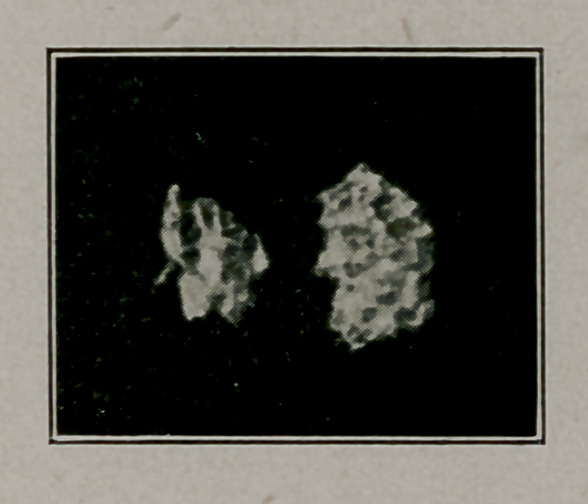


**Figure f8:**
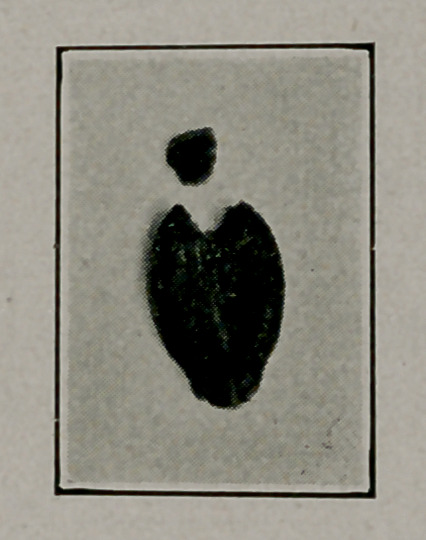


**Figure f9:**